# High-Resolution Phase-Contrast Tomography on Human Collagenous Tissues: A Comprehensive Review

**DOI:** 10.3390/tomography9060166

**Published:** 2023-11-27

**Authors:** Michele Furlani, Nicole Riberti, Maria Laura Gatto, Alessandra Giuliani

**Affiliations:** 1 Department DISCO, Università Politecnica delle Marche, Via Brecce Bianche 12, 60131 Ancona, Italy; m.furlani@pm.univpm.it; 2 Neuroscience Imaging and Clinical Sciences Department, University of Chieti-Pescara, 66100 Chieti, Italy; nicole.riberti@unich.it; 3 Department DIISM, Università Politecnica delle Marche, Via Brecce Bianche 12, 60131 Ancona, Italy; m.l.gatto@univpm.it

**Keywords:** phase-contrast tomography, soft tissues, collagen, human body, synchrotron radiation

## Abstract

Phase-contrast X-ray imaging is becoming increasingly considered since its first applications, which occurred almost 30 years ago. Particular emphasis was placed on studies that use this technique to investigate soft tissues, which cannot otherwise be investigated at a high resolution and in a three-dimensional manner, using conventional absorption-based settings. Indeed, its consistency and discrimination power in low absorbing samples, unified to being a not destructive analysis, are pushing interests on its utilization from researchers of different specializations, from botany, through zoology, to human physio-pathology research. In this regard, a challenging method for 3D imaging and quantitative analysis of collagenous tissues has spread in recent years: it is based on the unique characteristics of synchrotron radiation phase-contrast microTomography (PhC-microCT). In this review, the focus has been placed on the research based on the exploitation of synchrotron PhC-microCT for the investigation of collagenous tissue physio-pathologies from solely human samples. Collagen tissues’ elasto-mechanic role bonds it to the morphology of the site it is extracted from, which could weaken the results coming from animal experimentations. Encouraging outcomes proved this technique to be suitable to access and quantify human collagenous tissues and persuaded different researchers to approach it. A brief mention was also dedicated to the results obtained on collagenous tissues using new and promising high-resolution phase-contrast tomographic laboratory-based setups, which will certainly represent the real step forward in the diffusion of this relatively young imaging technique.

## 1. Introduction

Phase-contrast micro-computed tomography (PhC-microCT) is a non-destructive imaging technique that combines the high-resolution capabilities of micro-computed tomography with the contrast-enhancing properties of phase-contrast microscopy. This makes it a powerful tool for studying the internal structure of soft tissues and other low-contrast specimens.

On the one hand, microCT is a type of X-ray computed tomography that uses an X-ray beam to scan a specimen and create a three-dimensional (3D) image. In conventional tomography, X-rays are absorbed differently by different materials, so the image can be used to visualize the internal structure of the specimen [1].

On the other hand, phase-contrast microscopy is a technique that uses the difference in phase between light rays that pass through a sample to create an image. The phase shift can occur due to the density, structure, or composition of the sample [2]. This technique can be used to create high-contrast images of transparent or weakly absorbing samples, as the SIGNAL obtained by the phase analysis is up to three times higher than with the absorbing methods for soft tissues [3].

In this way, combining microCT and phase-contrast microscopy provides several advantages. First, phase contrast can be used to enhance the contrast of low-contrast samples, which can improve the image quality, or of image samples that are completely transparent to X-rays, such as soft tissues. Secondly, PhC-microCT is a non-destructive technique, so the sample can be imaged multiple times without damage in further analysis.

PhC-microCT has been used to study a wide variety of biological and medical specimens, including cells, tissues, organs, and whole organisms. It has also been used to study materials, such as plastics, ceramics, and metals.

In this review, the evolution of its deployment will be investigated, starting from the first setups and applications, with a particular focus on human collagen-based tissue studies, which represent the inclusion criteria that was adopted.

Collagen constitutes a family of approximately 30 proteins indispensable for establishing and maintaining the structural and functional integrity of various tissues throughout the human organism. Collagen type I prevails as the most abundant variant, forming fibrous networks that confer tensile strength and structural support to tissues, such as skin, tendons, and bones. It is noteworthy that the three-dimensional architecture and arrangement of these collagen networks are intricately tailored to meet the distinct requirements of different tissues [4].

The structural characteristics and mechanical properties of collagen fibers extend their influence beyond the general function of the tissue as a whole to affect the behavior of individual cells within this microenvironment. Collagen fibers offer a various array of topographical, biochemical, and mechanical cues that orchestrate cellular vital processes, such as proliferation, differentiation, migration, and apoptosis [5]. This mechanobiological interplay between cells and the surrounding collagen-rich extracellular matrix assumes a pivotal role in physiological phenomena, such as wound healing. However, deviations in the mechanical properties of the collagen network, leading to abnormal stiffness, can instigate pathological processes, including fibrosis, cancer, and metastasis [6,7].

Tissue-specific characteristics of collagen, resulting in variety and complexity, suggest that the investigation of its behavior, in both physiological and pathological conditions, requires the effort of gathering human samples, which provide a guarantee of the reliability of the results obtained.

Therefore, owing to the progress in imaging methodologies, the utilization of human biopsy specimens extends beyond diagnostic purposes. For instance, empirical data indicate that the stromal component assumes a pivotal role in the regulation of tumor progression, particularly after the initial stages of tumorigenesis. Furthermore, the stromal element significantly contributes to the determination of risk factors associated with the genesis of tumors [6,7].

Thus, PhC-microCT analysis could become fundamental for the comprehension of these events to foster and accelerate the discovery of new and effective therapeutic approaches.

## 2. Exploring the Capabilities of PhC-microCT in Different Setups

Starting from mid 1990s, several techniques were tested attempting to contemporarily obtain, in soft tissues, good signal-to-noise contrast and high resolution; the focus on these exploratory research studies was to evaluate the capabilities of each technique and compare it with the previous gold standard, also highlighting its advantages with respect to every other current method.

Phase-contrast X-ray computed tomography produced some of the most interesting results; indeed, the possibility of achieving good results has been proved by exploring the following three setups: the analyzer-based imaging, the X-ray grating interferometry, and the propagation-based phase tomography.

### 2.1. Analyzer-Based Imaging

**Figure 1:**
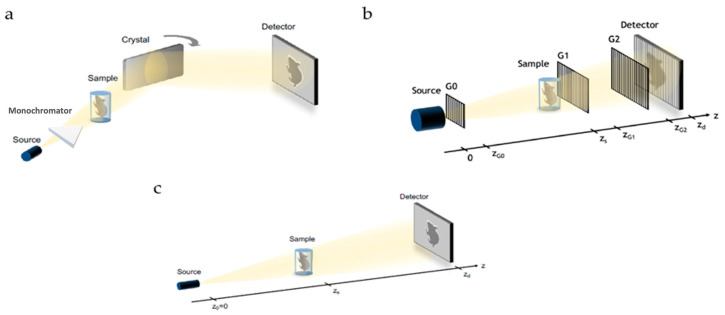
Schematics of most common phase-contrast setups: (**a**) analyzer-based, (**b**) grating interferometry, and (**c**) propagation-based. (Ref. [8], CC-BY 4.0).

Analyzer-based imaging (ABI) is a phase gradient-sensitive technique that uses highly selective analyzer crystals to filter the transmitted X-ray beam. The experimental setup for ABI entails the placement of a monochromator crystal preceding the sample and an analyzer crystal situated between the sample and the detector, according to the Bragg’s geometry (Figure 1a). In the absence of a sample, if the analyzer crystal is aligned to the same angle as the crystal creating the monochromatic beam, all the monochromatic incoming X-rays are completely diffracted. However, in the presence of a sample, the analyzer crystal functions as an angular filter for the monochromatic radiation emitted by the sample. This phenomenon arises because the X-rays refracted by the sample exhibit varying angles of incidence upon reaching the analyzer crystal. Consequently, when these X-rays impinge upon the analyzer crystal, the Bragg diffraction condition is met only within a limited range of angles. In contrast, if the scattered or refracted X-rays possess incident angles falling outside this specified range, they remain unreflected and do not contribute to the signal. The manipulation of the analyzer crystal’s tilt angle allows for the extraction of the refraction angle of the X-rays. Detectors that are commonly used include CCDs or CMOS, depending on the needs of the experiment and on the energy range of the radiation. ABI has several advantages, including its high sensitivity, low dose deposition to samples, and the fact that it has been successfully implemented not only at synchrotrons but also with laboratory sources. However, it also has some disadvantages, such as low beam stability (even at synchrotrons) and the need for long exposure times because of the low flux resulting after monochromatization. These characteristics make ABI a poor candidate for the transfer of conventional laboratory-based X-ray sources, as demonstrated by the very few works that have been completed in this direction. Overall, ABI is a promising technique for phase-contrast imaging, but it is not yet suitable for widespread use in clinical or research settings. Further research is needed to address the challenges of beam stability and low flux in order to make ABI a more practical imaging modality [8,9].

### 2.2. Grating Interferometry

X-ray grating interferometry (GI) is a phase gradient-sensitive technique that uses gratings to create periodic interference patterns. The experimental setup for GI comprises two gratings, denoted as G1 and G2, a detector, and, in the case of a conventional X-ray source, an additional grating denoted as G0, which serves as a collimator to generate multiple small X-ray sources. Typically, G1 is designed as a phase grating, while G2 functions as absorption gratings. G1 serves in beam splitting, creating periodic interference patterns that vary with distance, referred to as the “Talbot carpet” (Figure 1b). This interference pattern arises from the waves remitted through G1 and manifests as periodic peaks of intensity at characteristic distances. The detector in this setup comprising photon-counting pixel detectors could also be found, and is strategically positioned at one of these characteristic distances, typically where the intensity peaks are most pronounced. When a sample is introduced into an X-ray beam, these interference patterns become distorted due to various physical phenomena, including attenuation, refraction, and scattering. To extract valuable information from these distortions, an attenuation grating with the same periodicity as the original interference pattern is placed in front of the detector. This configuration translates the phase variations into intensity variations because the intensity peaks no longer align with the gaps between the bars of G2. To attain precise measurements of refraction angles and to characterize the attenuation and scattering properties, multiple acquisitions are conducted while adjusting the positions of the gratings. This comprehensive approach allows for the retrieval of detailed information regarding refraction, attenuation, and the scattering of X-rays, facilitating a more comprehensive analysis of the sample under investigation. GI has several advantages, including good sensitivity and the fact that it can be used with conventional X-ray sources. However, it also has some disadvantages, such as the need for complex and expensive experimental setups, especially for large fields of view. Furthermore, the acquisition time is still relatively long, and more studies are needed to reduce noise levels. Overall, GI is a promising technique for phase-contrast imaging, but it is not yet suitable for widespread use in clinical or research settings. Further research is needed to address the challenges of experimental setup complexity, acquisition time, and noise levels in order to make GI a more practical imaging modality [8,10,11].

### 2.3. Propagation-Based Imaging

Propagation-based imaging (PBI) is a phase gradient-sensitive technique that uses the spatial coherence of synchrotron X-rays to create interference patterns. The experimental configuration of PBI is similar to the conventional radiography one, with the only difference being that the distance between the detector and the sample depends on the desired level of the spatial resolution (Figure 1c). When the X-rays pass through the sample, they are refracted by the different materials in the sample. This refraction causes the X-rays to interfere with each other, creating interference patterns. The occurrence of these interference patterns becomes increasingly pronounced as the separation distance between the sample and the CCD or CMOS detector is extended. These interference fringes manifest as alternating white and black lines that outline the contours of the sample. The phase of the X-rays can be retrieved from the interference fringes using a technique called phase retrieval. PBI has several advantages, including its simple experimental setup, rapid acquisition time, and relatively low noise levels. However, PBI also has some disadvantages: It is not as sensitive as other phase-contrast imaging techniques, such as ABI. It is also not as well-suited for imaging large fields of view. It is the most widely used technique in synchrotrons, and it is becoming increasingly popular in laboratory settings.

It is necessary to retrieve the phase information to obtain the desired image. Numerous techniques have been devised and refined globally for this purpose, yet the cornerstone among them is the widely recognized “Paganin method” [12]. This method relies upon the application of the transport-of-intensity equation (TIE) under the paraxial approximation described by the following equation:

$$-k\frac{\partial I\left(x,y\right)}{\partial z}=\nabla_{⊥}\cdot [I\left(x,y\right)\nabla_{\phi }\left(x,y\right)]$$

where k is the wave number 2π/λ, x and y are the spatial coordinates in the plane perpendicular to the propagation axis z, I(x, z) is intensity distribution at the plane located at the propagation distance z, and ∇ is gradient operator over x. A comprehensive expansion of this equation, including its derivation and its application through various approaches, can be found in the highly detailed and well-crafted review authored by Zuo et al. [13].

## 3. Moving the Focus from the Experimental Setting to the Biological Result

PhC-microCT proved to be accurate and able to provide interesting insights on soft tissues, otherwise not obtainable with absorption-based settings. In particular, PhC-microCT has been recently employed to study collagen-based tissues in several skeletal districts, both in animal models and in humans, considering biopsies as well as in vivo pre-clinical and clinical settings.

While there is a vast literature of in vitro and in vivo studies (but limited to the use of animal models) on collagen-based materials, there are fewer studies that focus on human tissues. Only recently it has been understood that, since collagen is a very heterogeneous structure that links its in vivo behavior to the biomechanical performance of the anatomical site, it is extremely important to collect and discuss the first studies in the literature that are exclusively referred to human tissues.

Thus, in this review, the most interesting exploratory studies that focused on high-resolution phase-contrast tomography on human collagenous tissues were reported and discussed, dividing the study material according to the relevant anatomical area.

### 3.1. Breasts

A substantial amount of experimental and clinical data underlines the pivotal role played by the stroma in the progression of breast cancer, emphasizing the imperative need for further research endeavors in this domain. Specifically, the evaluations of collagen density and alignment emerge as particularly pertinent biomarkers in this context. Remarkably, the utilization of collagen alignment as a biomarker readily aligns with the assessment of other biomarkers, presenting a coherent approach to diagnostics. The significance of collagen alignment, the extracellular matrix (ECM) and diverse stromal cell populations in facilitating cellular invasion, connects these predictive factors to the underlying mechanisms governing cell invasion. A more profound comprehension of these intricate processes holds the promise of unveiling novel avenues for the diagnosis, prediction, and therapeutic intervention in breast cancer [14]. In particular, there is a recent and growing interest in methods of in situ discrimination of an aggressive ductal carcinoma (DCIS) compared to an indolent one. Sprague et al. [15] studied the collagen organization in the DCIS tumor microenvironment in relation to pathologic characteristics and patient outcomes; they found that multiple aspects of collagen organization around DCIS lesions are associated with a risk of recurrence. Thus, PhC-microCT imaging of collagen organization should be considered in the development of prognostic DCIS biomarker signatures.

The first phase-contrast investigations of the breast at synchrotron radiation facilities were performed in the year 2000 [16]. Afterward, Takeda et al. proved that phase-contrast imaging with an X-ray interferometer was a highly sensitive method to differentiate the inner soft tissue in breasts, detailing morphological structures that could be imaged without using contrast agents [17].

In the same year, the use of the diffraction-enhanced imaging (DEI) method was shown to provide better detailed visibility, necessary to achieve correct assessments and earlier detection of breast ductal lesions [18].

However, a relevant limitation to the clinical translation of the DEI method has been the need to use synchrotron radiation. In this direction, Faulconer et al. [19] developed a DEI prototype (DEI-PR) using a Tungsten X-ray tube laboratory source combined with traditional DEI crystal optics. Full-thickness human breast tissue specimens were imaged with the DEI-PR at 60 keV, but also with a synchrotron-based DEI and digital mammography systems to demonstrate the clinical utility of the laboratory-based prototype. Data analysis suggested that the DEI-PR system performed similarly to the traditional DEI system, demonstrating a significant step toward clinical translation of the DEI method for breast cancer applications.

The following year, using the PBI method, synchrotron-based images of various breast diseases (fibrocystic change, ductal carcinoma in situ, invasive ductal carcinoma, Paget’s disease) were obtained [20]. When compared to the standard histopathologic findings, these images showed relevant features for each disease, suggesting that synchrotron-based PBI has potential as a diagnostic tool in breast imaging.

However, we had to wait until 2011 to have the first phase-contrast mammography tests performed on patients, when, at the Elettra synchrotron, excellent results were obtained in terms of specificity and sensitivity through a dichotomization system able to recognize benignancy and malignancy in breast abnormalities [21].

Another step towards applying PhC-microCT in the clinical setting was made in 2014, when the feasibility of quantitative breast tissue characterization using grating-based phase-contrast tomography with polychromatic X-ray sources was demonstrated. Different breast specimens, including phyllodes tumors, fibroadenomas, and infiltrating lobular carcinomas, were imaged. Adipose, fibroglandular, and tumor tissues were analyzed in terms of phase-contrast Hounsfield units and compared to high-quality, high-resolution data obtained with monochromatic synchrotron radiation and provided promising results [22].

Meanwhile, other results of interest were obtained using synchrotron radiation as the X-ray source. Indeed, as the PBI phase-contrast technique requires a spatially coherent source and a sufficient object-to-detector distance, the effect of this distance on image quality was quantitatively investigated on a large breast surgical specimen at three object-to-detector distances (1.6, 3, 9 m). The obtained images were compared both before and after applying the phase-retrieval procedure. The study showed that, for phase-retrieved images, changing the object-to-detector distance did not affect spatial resolution; however, it boosted the signal-to-noise ratio [23].

### 3.2. Cardiovascular System

Cardiovascular disease stands as a predominant global cause of mortality. Consequently, there is an imperative demand for the development of innovative therapeutic strategies in this domain. Unfortunately, today, most experimental research is performed using young animals, while these diseases are often related to aging.

We know that the cardiac ECM consists predominantly of fibrillar collagen; this serves to uphold the integrity of myocardial tissues, enabling effective force transmission and the maintenance of myocyte geometry. Consequently, any disturbances in the regulation of collagen synthesis, subsequent deposition, associated modifications, and eventual degradation can exert deleterious effects on myocardial function. It is widely acknowledged that the aging heart is notably marked by a process of fibrotic remodeling [24].

However, the mechanisms responsible for disruptions of collagen synthesis are not fully understood not only in the presence of heart aging but also in the case of patients suffering from various cardiovascular diseases.

The microscopic analysis of the human heart and vascular tissue specimens plays a major role in enhancing our comprehension of cardiovascular diseases. Unfortunately, to date, only a limited number of investigations have managed to successfully generate three-dimensional images of the heart and major blood vessels. Consequently, given the scarcity of available specimens afflicted with intricate cardiovascular pathologies, it becomes paramount to handle these samples with utmost care, avoiding any damage or alteration of their inherent properties during the examination process. X-ray PhC-microCT is an imaging technique especially suited for soft tissue investigations because it is non-destructive and preserves the properties of specimens. The feasibility of PhC-microCT was recently tested for the structural investigation of heart specimens using infantile and fetal hearts without congenital diseases [25]. The images clearly showed the myocardial structure, the coronary vessels, and the conduction bundle, confirming that PhC-microCT X-rays can provide a fundamental contribution to the structural investigation of the heart affected by congenital diseases.

**Figure 2:**
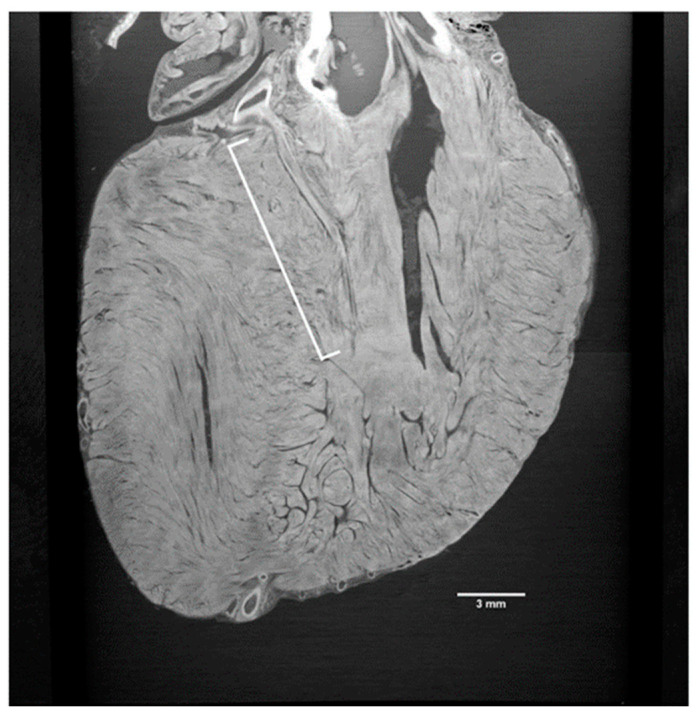
X-ray phase-contrast tomography image displaying the initial septal branch of the left anterior descending artery (LAD); here, a two-dimensional depiction of the heart is captured from a distinct angle. The image distinctly showcases the section of the first septal branch (highlighted by the white bracket) stemming from the LAD (CC-BY 4.0) [26].

Indeed, Tsukube et al. [26] were able to resolve, with synchrotron radiation-based PhC microCT, the 3D morphology of various cardiovascular tissue samples, including the thoracic aorta, ductus arteriosus, and cardiac conduction system. In particular, PhC microCT showed differences in 3D structures of the tunica media in an aortic dissection, 3D morphological changes in the transition from the ductus arteriosus to the descending aorta in a surgically excised sample of coarctation of the aorta, and it was also useful for examining abnormalities of the cardiac conduction system in congenital heart defects (Figure 2).

In a more recent study, the PhC-microCT technique was employed to analyze ascending aorta specimens obtained from patients diagnosed with an acute type A aortic dissection (ATAAD). Differences in the anatomy of the aortic wall were seen in the specimens of the aortic dissection, both with and without Marfan syndrome (MFS), as well as in normal aortas. The study found that the medial density values exhibited uniformity in the healthy aortas, while displaying significant differences in individuals with Marfan syndrome (MFS). Furthermore, the medial density was seen to be lower and exhibited distinct characteristics in individuals without MFS [27]. The results of this study indicate that there are structural differences between the aortic wall of an acute aortic dissection and a normal aortic wall. These differences are likely attributed to an uneven ratio of elastin to collagen.

PhC-microCT was also successfully used for the characterization of human coronary artery plaques. PhC-microCT and conventional absorption-based microCT were performed ex vivo to study 40 human coronary artery segments by using a synchrotron radiation source. Quantitative measurements of plaque components were compared among phase-contrast and absorption images. While PhC-microCT was able to discriminate several plaque components (calcifications, collagen, lipids, and smooth muscle cells), the absorption images were not able to discriminate lipids and smooth muscle cells. Thus, it was shown that PhC-microCT is also able to accurately characterize human coronary artery plaques, quantitatively assessing plaque components [28].

### 3.3. Auditory Apparatus

The auditory system contains numerous forms of collagen, including types I, III, and V, which are present in a healthy middle ear. Additionally, the cartilage of the middle and inner ear has been revealed to include minor cartilage collagen types IX and XI. The epithelial and endothelial basement membranes are the main locations where collagen type IV is typically found [29].

Conventional imaging methods used to detect collagen-based soft tissues in the ear can be laborious and may compromise the geometrical accuracy of certain structures because of the difficulty in discerning them, using absorption-based X-ray imaging, or because of extensive sample processing prior to imaging that may distort tissue geometry, like in case of histologic microscopy.

The synchrotron-based PhC-microCT has enabled the high-contrast visualization of the middle ear [30]; indeed, recently, it was shown that this technique can be successfully applied to image the ultrastructural characteristics of the human incudostapedial joint [31].

In this context, synchrotron radiation PhC-microCT was used to study the 3D microanatomy of the basal membrane (BM) and its attachment to the spiral ligament at the round window (RW) membrane in the human cochlea. A full understanding of this complex anatomy is essential to design and successfully insert innovative cochlear implants. Indeed, with PhC-microCT, it was found that, unlike other mammalian species, the human secondary spiral lamina is restricted to the most basal portion of the cochlea around the RW. It anchors the BM and may influence its hydro-mechanical properties [32].

With synchrotron PhC-microCT, the BM can be successfully imaged and measured in detail through its entire length to the helicotrema. Moreover, the method allows for the BM volumetric reconstruction without sectioning, decalcification, and staining [33]. Indeed, through PhC-microCT, it was determined that the distance between the end of the BM and the tip of the entire cochlea is clinically consequential. Additionally, a correlation between each patient’s individual cochlear duct length measured to the cochlea’s apical tip and their BM length was discovered [34].

**Figure 3:**
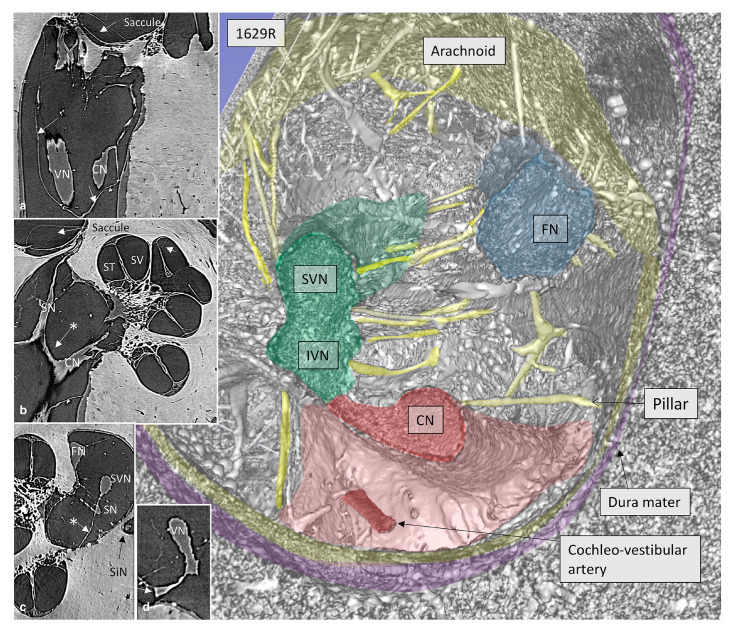
SR-PCI and 3D reconstruction show the nerve complexes in a left IAC surrounded by a continuous arachnoid sheet (arrows in inset a). The arachnoid is firmly attached to the dura wall (stained purple) except superiorly, where there is a subdural space. Inset b shows a section with a connection between the CN and SN (*) as well as arachnoid attachments to the IAC walls (arrows). In inset c, the arachnoid connections between the SVN and SN and fundus can be seen (*). The cochleo-vestibular artery (CVA) enters among the high-frequency nerve fibers and reaches the base of the cochlea. Other acronyms in the picture: ST, scala tympani; SV, scala vestibule (Ref. [35], CC-BY 4.0).

Moreover, the synchrotron PhC-microCT imaged the 3D anatomy of the human internal acoustic canal, including cranial nerve complexes, anastomoses, and arachnoid membrane invaginations (acoustic-facial cistern, an extension of the cerebellopontine cistern), without processing and decalcifying the samples (Figure 3). In detail, a complex system of arachnoid pillars and trabeculae was visualized, extending between the arachnoid membrane and cranial nerves. These arachnoid pillars were postulated to serve the function of providing structural support and stability to the cranial nerves within the internal acoustic canal. Additionally, it is conceivable that these structures may contribute to the modulation of local fluid dynamics within this anatomical region [35].

### 3.4. Neck and Odontostomatological Regions

The work of oral surgeons is largely responsible for the current understanding of the anatomy of the fasciae and fascial gaps of the maxillofacial and anterior neck regions. However, these descriptions have long been considered puzzling and overly complex. Fascia is a layer of connective tissue that surrounds and separates muscles, organs, and other structures. Fascial spaces are potential spaces that can be filled with fluid or air. The maxillofacial and anterior neck regions’ fasciae and fascial gaps are intricate and interconnected, and their anatomy is not fully understood. The utilization of modern imaging techniques, including magnetic resonance imaging (MRI) and computed tomography (CT), has contributed to the enhancement of our knowledge of the anatomical structures of the fasciae and fascial spaces in the maxillofacial and anterior neck areas. Despite these advances, there is still much that we do not know about the anatomy of these regions [36].

In this context, PhC-microCT was introduced with the objective of providing greater contrast and higher resolutions to resolve the details of this complex structure, both in physiological and pathological conditions.

In 2014, Zhang et al. [37] exploited its characteristics to evaluate the capabilities of this technique to illustrate the tri-layered composition of the esophageal wall without relying on a contrast agent and to visualize the mucosal layer without employing barium sulfate. This method enables the visualization of the demarcation and configuration of both healthy and tumor-afflicted tissue, highlighting the absence of the submucosal layer. These outcomes collectively furnish valuable diagnostic insights for the identification and assessment of esophageal carcinomas.

Another complex structure and its physiology have been explored by Bailly et al. in 2018 [38], who employed high-resolution synchrotron X-ray microtomography with phase-retrieval techniques to acquire the first ex vivo three-dimensional visual representations of human vocal-fold tissues across various magnifications. Human vocal folds are unique biological structures that can endure large deformations and vibrate at high frequencies. The capacity to exhibit this phenomenon can be attributed to the intricate three-dimensional and multiscale configuration of the subject, posing challenges for analyses using traditional imaging methodologies. The obtained images revealed the detailed structure of the vocal folds, including the geometry of the folds at rest and in a stretched phonatory-like position; the shape and size of the layered fibrous architectures; the orientation, shape, and size of the muscle fibers; and the set of collagen and elastin fiber bundles constituting these layers.

Moving the focus to the odontostomatological region, Iezzi et al. [39] presented a study on collagen remodeling around the abutment after implantation. It has been postulated that the presence of symmetric and well-organized connective tissues surrounding the longitudinal implant axis may potentially mitigate early bone resorption through the reduction of inflammatory cell infiltration. The existing body of research pertaining to the connective tissue around implants and abutments has primarily focused on two-dimensional analyses. Nevertheless, solely sophisticated three-dimensional characterizations can reveal how the connective tissue microarchitecture organizes itself and helps to identify new strategies to reduce inflammatory cell infiltration. It was demonstrated that synchrotron phase-contrast imaging is capable of studying 3D parameters that cannot be analyzed with histologic techniques but are important to define the 3D microarchitecture of the collagen bundles and the 3D structural complexity of connective tissues. Remaining in the same research field, Canullo et al. [40] performed an in-depth study on the impact of the implant macro- and micro-geometry on peri-implant connective tissues, most likely linked to the contact force at the interfaces between soft tissues and abutment.

### 3.5. Knee Joint and Surrounding Tendons

The study of degenerative joint diseases, such as osteoarthritis, is challenging because cartilage changes can only be visualized once there is joint space narrowing and thus patient cartilage loss. This has motivated the use of X-ray-based technologies that can provide an earlier detection of cartilage damage. Osteoarthritis (OA) is a leading cause of disability worldwide and has a high socioeconomic burden. It is a potential target disease for phase-contrast imaging because its diagnosis requires the analysis of both soft tissue, such as cartilage, and bony detail. Initial observations have shown that a high-quality imaging of cartilage structure is feasible with PhC imaging [41].

The first results were obtained with an analyzer-based phase-sensitive imaging (ABI) setup which generated high-quality images of both soft and bone tissues in the ankle and foot region [42]. However, these studies have been limited to planar (projectional) imaging or have focused on very small samples (generally in the submillimeter range). With their consequent work [43], Li et al. extended the results obtained in the planar mode to a three-dimensional level, focusing on a whole knee joint and obtaining promising results concerning image contrast and resolution. The following year, Coan et al. [44] carried out a study to visualize the internal architectural properties of the cartilage matrix in relatively large human patella cartilage samples. Specifically, the research group wanted to determine whether the obtained level of structural detail was sufficient to differentiate osteoarthritic from normal samples in analogy to histopathological criteria. In the case of osteoarthritis, histopathological criteria include the loss of cartilage, the formation of bone spurs, and the inflammation of the joint capsule. They found that ABI was able to visualize the internal architectural properties of the cartilage matrix in relatively large human cartilage samples. They also found that the level of structural detail obtained with ABI was sufficient to differentiate osteoarthritic from normal samples, in analogy to histopathological criteria. These findings suggested that ABI had the potential to be a valuable tool for the early diagnosis and treatment of osteoarthritis. In the wake of these promising results, Annie Horng et al. [45] broadened the perspectives evaluating the feasibility of high-resolution tomographic X-ray single-distance propagation-based imaging (PBI) for the depiction of soft tissues in whole human knees, with a focus on hyaline cartilage as it is often damaged in osteoarthritis. The method was compared with conventional computed tomography (CT), synchrotron radiation absorption-based CT, and magnetic resonance imaging (MRI). This study found that the PBI setting was able to visualize soft tissues in whole human knee joints with good contrast and resolution. In particular, it revealed that PBI was promising not only for the evaluation of the human knee joint, with relevant information about the cartilage, but also for the analysis of the underlying subchondral bone as well as their morphologic changes in osteoarthritic conditions.

**Figure 4:**
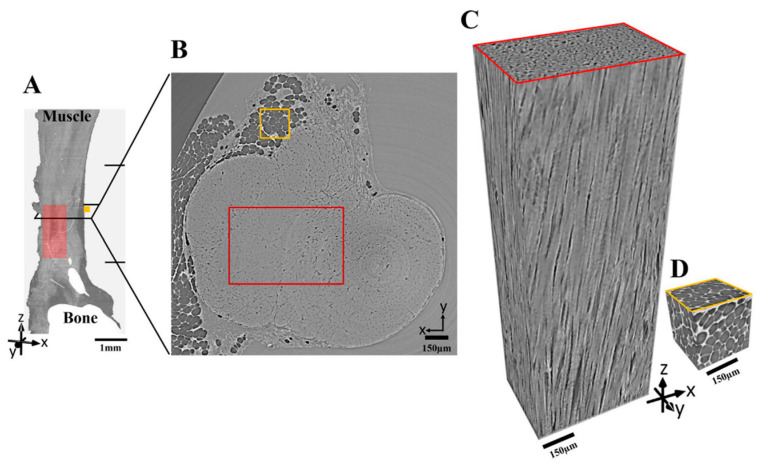
The areas of interest selected for the image quality analysis are as follows: (**A**) Volume rendering of the entire imaged tendon is presented, following the reorientation and combination of three consecutive image stacks acquired using the HNAM setup. The field of view for each scan is delineated by black horizontal lines on the right side of the image. (**B**) A cross-sectional view of the tendon is displayed, specifically within the central region identified in (**A**) through the black rectangle. (**C**) Within the red square outlined in (**A**,**B**), a volume rendering illustrates the organization of tendon fibers. (**D**) Similarly, within the yellow square delineated in (**A**,**B**), a volume rendering showcases the three-dimensional configuration of the adipose cells enveloping the tendon. (Ref. [46], CC-BY 4.0).

In the effort to raise the imaging quality, Pierantoni et al. [46] investigated the effects of sample preparation and imaging conditions on the resulting image quality of Achilles tendon collagen fibers (Figure 4). Four types of sample preparations were considered (fresh frozen, fixed, embedded, and stained) and two imaging setups (sub-micrometric and micrometric final pixel sizes). The image quality was assessed using four quantitative parameters that were defined and calculated using customized scripts (spatial resolution, fiber-to-matrix contrast ratio, uncertainty of the threshold-based segmentation of fibers and matrix, gradient sharpness at the edges). Their results showed that conventional invasive sample preparations, such as fixation and embedding, did not improve image quality. In fact, these procedures often degrade image quality by introducing artifacts. Fresh frozen samples, on the other hand, yielded high-quality images that effectively captured the intricate three-dimensional arrangement of tendon fibers, closely resembling their natural configuration. These findings suggest that fresh frozen samples are the preferred sample preparation method for imaging tendon collagen fibers as it preserves the native structure of the fibers and minimizes the introduction of artifacts.

From the same research group, the following year, Einarsson et al. [47] investigated the feasibility of synchrotron radiation-based PhC-microCT for the imaging of the human meniscus. Quantitative parameters pertaining to both fiber orientation and crimping were systematically assessed as prospective indicators of tissue degeneration. PhC-microCT was able to visualize the human meniscus with good contrast and resolution and the images were able to show the individual collagen fibers and the crimping pattern of the fibers. The study also found that the quantitative parameters related to fiber orientation and crimping were significantly different between healthy and degenerate menisci. This suggests that these parameters could be used as potential markers of tissue degeneration.

### 3.6. Lungs

The lung matrix is made of collagen and elastin fibers, intertwining with glycosaminoglycans (GAGs), fibronectin fibrils, proteoglycans (PGs), and water. To date, our knowledge of ECM composition in the lung is still in progress because some variants of these common matrix structural proteins have not yet been characterized [48]. The primary means of acquiring structural data on the fine structure of the human lung has predominantly been the utilization of stereological techniques applied to consecutive sections. However, the aforementioned approaches suffer from inaccuracies due to the use of 2D images that are not contiguous. These inaccuracies can be overcome by analyzing three-dimensional micro-CT images of the never-sectioned specimen, obtaining an improved accuracy, and increased spatial resolution, which allows for the visualization of smaller structures and reduced sample preparation time. First attempts were carried out by Litzlbauer et al. [49], focusing on acini characterization, and establishing that the dimensions they obtained agreed reasonably well with the published histology-based estimates. Consequently, micro-CT can provide such data in a more routine manner to quantitatively examine variation within a lung as well as between lungs.

Moving on to a lower scale, the lung’s microanatomy exhibits intricate patterns of branching, leading to the formation of complex structures. This makes it difficult to interpret tissue sections.

For instance, in several types of pulmonary hypertension, such as alveolar capillary dysplasia with misaligned pulmonary veins (ACD/MPV), the presence of intrapulmonary bronchopulmonary anastomoses (IBAs) has been documented. IBAs are abnormal connections between the bronchi and the pulmonary arteries. They are thought to be formed in response to increased pulmonary vascular resistance. IBAs can improve blood flow to the lungs, but they can also cause complications, such as bleeding and infection. ACD/MPV is a rare condition that is characterized by the abnormal development of the pulmonary vasculature. It is associated with a high risk of pulmonary hypertension and death. The study of IBAs is important for understanding the development of pulmonary hypertension and for developing new treatments for this condition. In 2019, Norvik et al. [50] used synchrotron-based phase-contrast micro-CT and subsequent 3D analysis in conjunction with a standard histological evaluation to confirm the presence of IBAs in ACD/MPV, defining a correlation between them, and demonstrating the advantages of the technique for the characterization of the pulmonary microvascular anatomy and pathology.

### 3.7. Ovaries and Uterus

A comprehensive understanding of the human ovary and uterus morphologies and physiologies is essential for the development of new diagnostics and reproductive technologies. This could be achieved by a clear and improved visualization of these organs’ structures and therefore by developing a methodology for comparing tissue fragments before and after freezing [51,52].

In this context, Pascolo et al. in 2019 [53] presented their work with the first imaging of human ovarian tissue by the use of synchrotron radiation propagation-based PhC-microCT, comparing their images with those acquired on the same samples with a conventional laboratory microCT. They assessed that the proposed approach could help to unravel important aspects of human ovarian micro-anatomy (follicles and vessels’ 3D distribution), helping in fertility preservation research. Indeed, collagen is a vital component of the blood vessels: they may undergo remodeling in response to structural modifications in these proteins.

From a pathological perspective, uterine leiomyoma (often known as uterine fibroids) is the prevailing non-malignant neoplasm of smooth muscle in the female pelvic region, coming specifically from the myometrium. The phenomenon is attributed to fibrosis, with a large production and disruption of the extracellular matrix. This gives rise to a substantial burden of illness among a considerable portion of women of a reproductive age, impacting their reproductive health and exerting repercussions on the outcomes of pregnancies in the affected individuals. The utilization of PhC-microCT has been employed for the purpose of imaging and performing quantitative morphometric analysis on both healthy myometrium and pathologic leiomyomas [54]. Modifications in the 3D structural network of this ECM protein have been evidenced in pathological conditions, in terms of changes in its secondary structure and spatial organization of bundles.

The same research group, in a following study [55], through a multimodal approach including PhC-microC, Fourier transform infrared imaging spectroscopy (FTIR), and transmission electron microscopy (TEM) analyzed the microstructural collagen characteristics and assessed the impact of eicosapentaenoic (EPA) and docosahexaenoic (DHA) omega-3 fatty acids on collagen reduction.

### 3.8. Other

In this concluding paragraph, studies that met the inclusion criteria of this review have been enclosed, focusing on human collagenous tissues analyzed with synchrotron PhC-microCT on anatomical districts which have not yet encountered many cases in the literature employing this technique.

#### 3.8.1. Hand and Its Vascularization

Solé Cruz et al. [56] studied the arterial anatomy of the hand and fingers in human cadavers for anatomical purposes. They focused on the vascularization and innervation of the hands by employing different resolutions to observe the structure of the whole hand and for the detail of the medial neurovascular bundle of the third finger at a higher resolution. The efficacy of this technique has been observed in facilitating the precise noninvasive examination of formations with varying sizes, widths, thicknesses, and histological origins.

#### 3.8.2. Stomach

Gastric cancer is a type of malignant tumor with high prevalence and mortality rate worldwide [57]. Early detection and diagnosis remain one of the key approaches to improve the prognosis of patients with gastric cancer. Tang et al. [58] investigated both the mucosa plane and cross-sectional specimens, firstly observing a fibrous scar at the bottom of an ulcer, the shape and boundary of the annular dike, and a penetrating area, measuring only 2 mm of the annular dike. All of the above could provide useful information for the classification of gastric cancer. The imaging of the cross-sectional specimen showed the three-layer structure of the gastric wall and depicted the infiltration of cancerous tissue in it, which may be helpful in the T-staging of gastric cancer.

## 4. Challenges and Perspectives

**Figure 5:**
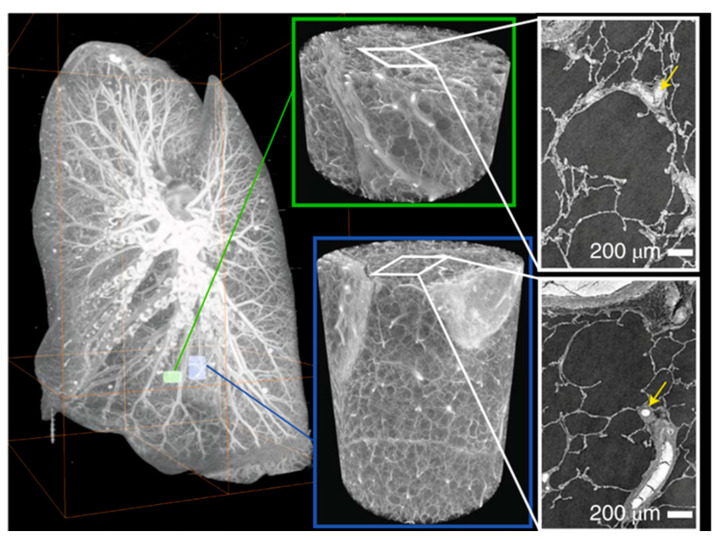
Maximum-intensity projection of a whole human lung with two randomly selected VOIs imaged at a resolution of 2.45 µm per voxel shown in green (VOI1) and blue (VOI2). Three-dimensional reconstructions of the two high-resolution VOIs are shown with 2D slices in the insets. In the 3D high-resolution VOI, the fine mesh of pulmonary blood vessels and the complex network of pulmonary alveoli and their septa can be seen. Yellow arrows denote occluded capillaries in 2D slices (Ref. [59], CC-BY 4.0).

Three-dimensional imaging of human intact organs containing large extensions of collagenous tissues is, to date, a challenging goal of biomedical imaging. In this direction, hierarchical phase-contrast tomography (HiP-CT) was developed using the European Synchrotron Radiation Facility (ESRF)’s Extremely Brilliant Source (EBS). Combining the X-ray phase propagation method to the spatial coherence of the ESRF-EBS, non-destructive 3D scans with hierarchically increasing resolution were realized in whole human organs. For instance, an intact upper right lung lobe, acquired from the autopsy of a 54-year-old male patient who died from COVID-19-related ARDS, was imaged. The lung was imaged at 25 μm per voxel, showing high-intensity regions in the lung periphery, consistent with patchy lung consolidation described using a conventional CT. At 6 μm per voxel, the loss of normal alveolar architecture was detected, with secondary pulmonary lobules displaying greater parenchymal deterioration than others. Finally, at 2.45 μm per voxel, imaging the more affected secondary pulmonary lobule, cavitation of lung parenchyma, alveolar obstruction, thickening of septa between adjacent alveoli, and blood capillary occlusion with adjacent cellular infiltrates were detected. These results indicated that HiP-CT can reproduce the microstructural findings observed in COVID-19 lung biopsies, investigating large tissue volumes with access to structures at different length scales [59] (Figure 5).

In a similar vein and leveraging HiP-CT at ESRF, Xian et al. [60] capitalized on the ability to separate sample size and resolution while retaining a strong ability to detect the microstructures of soft and/or partially dehydrated tissues. They successfully generated a complete ex vivo reconstruction of a human left lung, employing isotropic voxel sizes of 25.08 μm, as well as localized zooms ranging from 6.05–6.5 μm to 2.45–2.5 μm in voxel sizes. The researchers directed their attention towards the specific environment utilized for conducting lung imaging inside the framework of this experimental configuration.

Another notable challenge centers around the development of high-resolution laboratory-based implementations of phase-contrast imaging, distinct from the synchrotron-based implementations discussed thus far. The limited number of synchrotrons globally, coupled with the competitive procedures for securing beam time, restricts access and the feasibility of conducting longitudinal studies in this domain.

Different X-ray phase-contrast tomography systems, appropriate for usage in non-synchrotron laboratory settings, are nowadays explored, focusing on requirements in terms of temporal and spatial coherence. The aforementioned imaging techniques, namely propagation-based imaging, grating interferometry, edge illumination, or Zernike phase contrast, are commonly employed. It is worth noting that the beam used in these techniques is typically conical in nature, as opposed to the planar beam utilized in synchrotrons. Some excellent review papers [61] provided detailed overviews of these existing phase-contrast imaging techniques.

In particular, in Zernike phase contrast, the sample is subjected to a focused X-ray beam using a condenser ring. After traversing the sample, the phenomenon of diffraction takes place. Subsequently, the beam is re-concentrated by employing a zone plate, while a phase ring is employed to introduce a reduction in amplitude and a predetermined phase shift to the segment of the beam that did not undergo diffraction. The beam exhibits comparable intensity but exhibits a distinct phase shift in relation to the diffracted beam. The resulting interference between the two beams generates fluctuations in intensity on the detector, causing edge enhancement in the images. Consequently, phase-retrieval techniques utilize a specialized filter function to transform the contrast of edges in images into contrast within specific areas [62]. The Zernike phase-contrast technique has the ability to obtain spatial resolutions at or below the lower micrometer scale. However, its usefulness to preclinical and clinical studies is generally hindered by the limited field of view, typically restricted to tens of micrometers.

Indeed, the utilization of X-ray phase-contrast tomography in non-synchrotron settings has certain difficulties. Beams generated with commercially available conventional X-ray tubes exhibit very weak spatial and temporal coherence as a result of their large focus spot sizes and broad energy spectra, respectively. Furthermore, non-ideal experimental conditions must be tolerated, which also include environmental vibrations and not-fast-enough scan times due to the relatively low flux emitted with the X-ray tubes.

Ultimately, due to its high spatial resolution and contrast capabilities, laboratory-based X-ray phase-contrast tomography may evolve into a valuable instrument for regular quality assurance purposes of human collagenous tissues. However, according to contemporary scholarly research, it is recommended to address the persisting limitations associated with lengthy scan durations and the limited field of vision [63].

Nevertheless, this review widely showed that synchrotron-based phase-contrast computed tomography can resolve collagen tissues using 3D images in several body regions, and in clinical contexts.

From another perspective, it could also be interesting to observe the development of new imaging processing techniques, as this is also occurring for images obtained through other acquisition techniques [64,65]. Their introduction could improve the quality of the final image before undergoing segmentation and analysis.

In this direction, it was hypothesized that deep learning semantic image segmentation can better identify and classify collagen tissues with respect to the conventional thresholding methods. Indeed, neural networks and deep learning allow to quantify structures in phase-contrast images, which are otherwise not distinguishable; collagen bundles can be identified by their orientation and not only by their physical densities, similarly to conventional thresholding segmentation methods [66].

In fact, localized changes in the orientation and connectivity of collagen bundles can determine changes in local strains and mechanical functions, with consequent pathophysiological implications (fibrosis, inflammatory diseases, tumor growth, etc.) [67].

In conclusion, this review showed that the exploitation of phase-contrast tomography for the investigation of human collagenous tissue physio-pathologies produced encouraging outcomes. This technique is persuading different researchers to approach it, and is supported by optimized phase-retrieval algorithms as well as an increasing computational power, which is enabling a widespread use and increasing the confidence researchers have with this technique.

## Data Availability

Not applicable.
